# Outdoor exposure and vitamin D levels in urban children with asthma

**DOI:** 10.1186/1475-2891-12-81

**Published:** 2013-06-12

**Authors:** Sonali Bose, Patrick N Breysse, Meredith C McCormack, Nadia N Hansel, Robert R Rusher, Elizabeth Matsui, Roger Peng, Jean Curtin-Brosnan, Gregory B Diette

**Affiliations:** 1 Johns Hopkins University, Baltimore, MD 21205, USA; 2 University of South Carolina, Columbia, SC, USA

**Keywords:** Asthma, Vitamin D, Outdoor, Ultraviolet, Exposure

## Abstract

**Background:**

The inner-city pediatric population in the United States has a disproportionate burden of asthma. Recent attention has focused on the immunomodulatory role of vitamin D, which may be protective against disease morbidity. As the primary determinant of vitamin D status in humans is exposure to sunlight, we aimed to determine if 25-OH vitamin D levels in urban preschool children with asthma were low, influenced by time spent outdoors, and associated with asthma morbidity.

**Methods:**

Serum 25-OH vitamin D levels were measured at baseline in a cohort of 121 inner-city children ages 2–6 years with asthma in Baltimore, MD. Participants were followed longitudinally at 3 and 6 months to assess time spent outdoors, asthma symptoms through questionnaires and daily diaries, and allergic markers.

**Results:**

In a predominantly black population of preschool children, the median 25-OH vitamin D level was 28 ng/mL (IQR 21.2-36.9), with 54% of the children below the traditionally sufficient level of 30 ng/mL and 7.4% in the range associated with risk of rickets (< 15 ng/mL). The median time spent outdoors was 3 hours/day (IQR 2–4), and greater time spent outdoors was not associated with higher vitamin D levels. 25-OH vitamin D did not show seasonal variation in our cohort (p = 0.66). Lower 25-OH levels were correlated with higher IgE levels.

**Conclusions:**

Urban African-American preschool children with asthma have high rates of vitamin D insufficiency, and increased outdoor exposure is unlikely to correct these low 25-OH vitamin D levels. Repletion in this population may require dietary supplementation.

## Introduction

Asthma morbidity is strikingly high among inner-city children in the United States, especially among African-Americans [1,2]. While the reasons for this disparity are not entirely clear, several risk factors, including an urban environment enriched with air pollutants and unique allergens, lower socio-economic status, and poor diet, may predispose such children to more severe disease [2].

Of these factors, serum 25 hydroxy (OH) vitamin D levels are disproportionately low in such populations [3-5] and may help to explain higher rates of asthma morbidity. Natural vitamin D production is determined by the UV component of sunlight which permits its cutaneous synthesis, and, given sufficient exposure to UV rays, results in currently accepted serum 25-OH D levels for humans. Cutaneous vitamin D synthesis, however, can be limited by darker skin pigmentation (as increased melanin competes for UV radiation), sunscreen use, clothing, and seasonal, atmospheric, and latitudinal factors that limit the availability of UV light [6]. Furthermore, dietary sources of vitamin D are limited to a few fortified foods [7], with African-American children found to have comparatively low D-rich intake [8]. Thus, while Americans generally spend the greater portion of their time indoors [9], inner-city children, especially African-Americans, may have additional risks for vitamin D deficiency due to risk factors that include having darker skin and a diet inadequate to compensate for deficiencies in production.

Furthermore, while vitamin D’s role in protecting against growth retardation, skeletal deformities, rickets, and other bony diseases of childhood has been well established [10], emerging literature has supported its role in various chronic diseases, including asthma and allergy [11]. In children with asthma, those with lower serum 25-OH D have a higher risk of hospitalization [12,13], greater use of anti-inflammatory medications [12,14], more airway hyper-responsiveness [12,13], and in adults, decreased sensitivity to corticosteroids [15]. Furthermore, *in vitro* studies have demonstrated that vitamin D may play an immune-modulatory role in human airway tissue through innate, adaptive immune pathways [16]. Notably, a recent trial of vitamin D supplementation in children with asthma did demonstrate improved asthma control such as reduced rates of exacerbations in those who received supplementation [17], suggesting that augmentation of 25-OH D levels could hold promise for future asthma health interventions.

Of studies that have examined the impact of vitamin D status on childhood asthma severity, there has not been much attention to black, inner-city populations, where rates of asthma-related hospitalizations are 2–3 times the national average (48 vs. 17 per 10,000) [18,19]. To better understand its significance in an inner-city pediatric population, we studied vitamin D status in a cohort of predominantly African-American preschool children with asthma in urban Baltimore. We sought to characterize the prevalence of 25-OH vitamin D insufficiency, investigate if 25-OH D levels are related to time spent outdoors, and prospectively evaluate the effect of vitamin D status on asthma morbidity.

## Subjects and methods

### Study design

The Baltimore Indoor Environmental Study of Asthma in Kids was a longitudinal study designed to investigate the role of indoor pollutants and allergens on asthma. One hundred and fifty children with asthma ages 2 to 6 years of age were recruited. Serum 25-hydroxy vitamin D levels were measured at baseline, and health outcome information was collected from caregivers at baseline, 3 and 6 months. Procedures followed were in accordance with the ethical standards of the Johns Hopkins Medical Institutional Review Board, which approved the study, and caregivers of participants provided informed consent prior to enrollment.

### Participants

Participating children were recruited from health systems providing care to most residents of East Baltimore (MD). Enrollment began September 2001 and the last patient follow-up was completed in April 2004. Potential subjects were first identified if they had a health care encounter assigned an ICD-9 (International Classification of Disease, 9th Revision) code of 493. X in the previous 12 months. They were confirmed as having asthma if they 1) had a physician diagnosis of asthma, and 2) had symptoms and/or medication use for asthma in the previous six months. All subjects maintained residence within one of nine contiguous ZIP codes within East Baltimore, which, based on 2000 US Census data, represents a geographic region that is >99% urban. We excluded children with significant co-morbid conditions.

### Laboratory evaluation

Blood drawn from children at the baseline evaluation was sent to the Johns Hopkins General Clinical Research Center labs for measurement of total 25-hydroxy vitamin D by radioimmunoassay (RIA) kit (DiaSorin, Stillwater, MN). The intra- and inter-assay coefficients of variation for vitamin D were 5.19 and 7.90, respectively. The preferred indicator of vitamin D status and the predominant circulating form is 25-OH D [20]. Serum 25-OH levels were stratified in the following brackets: ≤ 20, 21–30, and >30 ng/mL, and defined as deficient, insufficient, and sufficient, respectively, according to previously established guidelines for bone health [20,21] (in the absence of consensus regarding appropriate levels for endocrine and extra-endocrine health) and levels below 15 ng/ml considered to impart higher risk for rickets [10]. Serum IgE levels, were also measured (ImmunoCAP, Phadia, Uppsala, Sweden).

### Clinical evaluation

At an initial visit, children underwent allergy skin testing with a panel of 14 aero-allergens: American and German cockroach, dust mite mix, cat dander, dog hair/dander, mouse epithelia, rat epithelia, three pollens (Eastern Oak mix, grass mix, ragweed mix) and four molds (Helminthosporium, Alternaria, Penicillum, and Aspergillus). Atopy was defined as at least one positive skin test result, defined as a wheal size greater than a glycerin control of ≥ 2 mm.

Evaluation of asthma morbidity was obtained at each time point from a health questionnaire completed by caregivers, which included close-ended questions derived from standard asthma symptoms questions used in prior studies of inner-city children with asthma [22]. We asked caregivers about acute health care use in the prior 3 months (emergency department visits, unscheduled doctor visits, and hospitalizations). We also collected information regarding the number of days (0–14) each child had the following symptoms in the previous 2 weeks: 1) wheezing, cough or tightness in the chest; 2) the need to slow down or stop activities because of asthma symptoms; 3) wheezing so badly that the child could only speak one or two words at a time between breaths; 4) cough without a cold or flu; 5) symptoms with exercise; and 6) nocturnal symptoms. Outdoor exposure was quantified both from questions regarding each child’s average number of daily outdoor hours across each season and a prospective daily time-activity diary completed by caregivers during the study.

### Statistical analysis

The 25-OH D levels in this population were summarized with means and proportions. Differences in vitamin D levels were compared across 4 seasons using the Kruskal-Wallis test for non-normally distributed continuous variables. Symptom-free days in a 2-week period were calculated at baseline, 3, and 6 months by subtracting the number of days of reported asthma symptoms from 14 days. Negative binomial regression with generalizing estimating equations [23] accounting for repeated measures was used to determine the relationship between baseline 25-OH D levels and the mean number of days of asthma symptoms across three time points. We constructed multivariate models accounting for age, gender, and socioeconomic factors. P values of less than 0.05 were considered statistically significant. All analyses were performed using StataSE software (Stata 11.0, College Station, Tx).

## Results

We had sufficient serum to analyze 25-OH D for 121 of the 150 subjects. This subset did not differ demographically from the parent cohort. The children were predominantly African-American (91%) and from households of low economic status. The majority of children (69%) was atopic and had active asthma symptoms, with 62% meeting criteria for persistent asthma (Table 1).

**Table 1:** Baseline characteristics of study participants by serum 25-OH vitamin D concentration

**Characteristic**	**All children (n = 121)**	**Vitamin D level**			**P value**
		**Deficient (<= 20) n = 28 (23%)**	**Insufficient (21–29 ng/mL) n = 37 (32%)**	**Sufficient (> = 30 ng/mL) n = 56 (45%)**	
Age (y) mean (range)	4.4 (2–6)	4.5 (2–6)	4.9 (2–6)	4.1 (2–6)	0.06
Male sex [n (%)]	73 (60)	17 (61)	23 (62)	33 (59)	0.84
Black race [n (%)]	109 (90)	28 (100)	33 (89)	48 (86)	0.05
BMI (kg/m^2^)	17.8 (4.1)	17.9 (4.7)	18.7 (5.7)	17.2 (2.1)	0.85
Public health insurance [n (%)] (n = 118)	106 (90)	21 (78)	34 (94)	51 (93)	0.07
Household income less than $15,000/yr [n (%)] (n = 62)	34 (55)	9 (64)	10 (59)	15 (48)	0.49
Caregiver a high school graduate [n (%)]	76 (63)	19 (68)	22 (59)	35 (63)	0.62
Atopic [n (%)] (n = 120)	83 (69)	21 (78)	22 (59)	40 (71)	0.79
Number of positive skin tests (mean ± SD) (n = 120)	2.71 ± 2.91	2.89 ± 2.39	2.38 ± 2.77	2.84 ± 3.25	0.66
Acute care in the past 3 months [n (%)]					
Emergency department visit	28 (23)	6 (21)	9 (24)	13 (23)	0.89
Hospitalization	4 (3)	0 (−)	1 (3)	3 (5)	0.19
Unscheduled doctor visit	22 (18)	6 (21)	6 (16)	10 (19)	0.75

### Levels of vitamin D measured in this population

**Figure 1:**
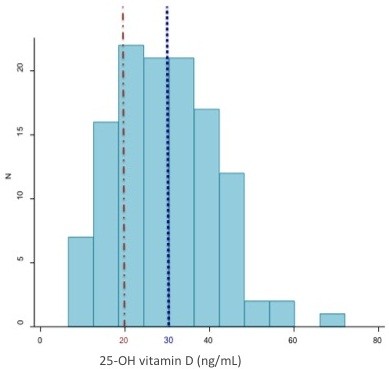
**Distribution of serum 25-OH vitamin D levels.** Histogram of 25-OH vitamin D levels. The median 25-OH D level in this cohort was 28 ng/mL (IQR 21–37). The majority (54%) of children had levels below current guidelines of sufficiency (30 ng/mL) (dotted line), with almost a quarter (23%) in the deficient (≤ 20 ng/mL) range (dashed line).

The median (IQR) serum 25-OH D level was 28 ng/mL (21–37). Fifty-four percent of subjects were found to be insufficient or deficient, with levels below the accepted sufficient cut-off of 30 ng/mL, and 23% in the deficient range with serum levels of 20 ng/mL or below (Figure 1). About 7.4% of all subjects had 25-OH D levels in the range associated with the risk of rickets (< 15 ng/mL). Vitamin D levels for the small number of whites tended to be higher despite equivalent socio-economic status (white vs. African-American: median 38 vs. 28 ng/mL, p = 0.07).

### Effect of hours spent outdoors on 25-OH vitamin D levels

**Figure 2:**
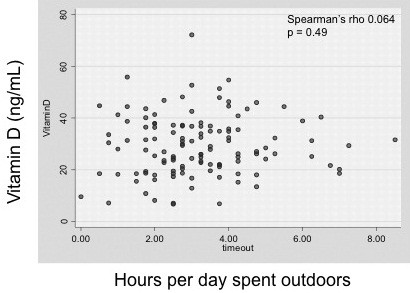
**Relationship between daily time spent outdoors and serum 25-OH vitamin D levels.** A scatterplot of the average hours per day spent outside vs. serum 25-OH vitamin D shows no correlation between the two. The median time spent outdoors was 3 hours/day (IQR 2–4).

On average, children in our cohort spent 3 out of 24 hours outdoors daily (IQR 2–4 hours). Time spent outdoors did not differ by gender (girls vs. boys: mean 3.1 vs. 3.2 hours). There was no association between the reported average daily hours spent outdoors and baseline 25-OH D levels (p = 0.49) (Figure 2). It is worth noting, though, that none of the 121 children who spent more than 5 hours per day outside was 25-OH D deficient.

### Effect of season on 25-OH vitamin D levels

**Figure 3:**
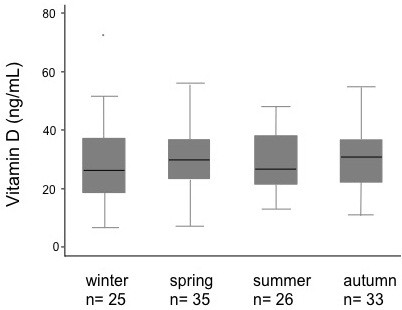
**Seasonal variation in 25-OH vitamin D.** Box-plots of 25-OH vitamin D by season. Levels of serum 25-OH D did not vary with respect to the season in which they were drawn (p = 0.66).

Samples of blood were distributed evenly with regard to the season in which they were drawn (winter, 25; spring, 35; summer, 26; fall, 33). There was no observed difference in serum 25-OH D levels among the seasons (p = 0.66) (Figure 3). When stratified by season of assessment, there was no association between serum 25-OH D levels and the outdoor time spent by each child in that same season (data not shown).

### Effect of vitamin D levels on asthma indicators

There was a modest, statistically significant inverse correlation between 25-OH D levels and total IgE concentrations (Spearman’s rho = −0.24; p = 0.03). There was no association between 25-OH D levels and atopic status as defined by positive skin testing (p = 0.87), nor was there a significant correlation between 25-OH D level and the number of positive skin tests (Spearman’s rho = −0.0026; p = 0.98). Additionally, there was no association between 25-OH D levels and acute health care visits (Table 1). In multivariate regression models, controlling for age, sex, and education level, asthma symptoms (cough without a cold or flu, cough/wheeze/chest tightness, and occurring with exercise) tended to be reduced with higher 25-OH D levels, though the associations were not significant.

## Discussion

This study demonstrated that 25-OH vitamin D insufficiency is present in the majority of a cohort of urban, preschool-aged inner-city children with asthma. These findings extend those seen in heterogeneous populations of older children [12,13] to a population of predominantly African-American children 2–6 years of age. Even at this early preschool age, 25-OH D levels were already strikingly below the recommended range, as defined by current guidelines for bone health. Over half (55%) were either insufficient or deficient, and a disturbing proportion of children (7%) in our cohort were at the extreme end of deficiency, with levels as low as those seen with rickets (< 15 ng/mL). The implications of these findings may be even greater in younger cohorts, as it is during early childhood when considerable lung growth is still occurring, and when the respiratory system may be particularly sensitive to nutritional and environmental signals that induce changes in lung function [24].

Our findings are comparable to the NHANES 2001–2004 study of US children (age 1–21 years), where nine percent of all children had levels less than 15 ng/mL, and sixty-one percent had levels in the range between 15 and 29 ng/mL [5]. In fact, within our defined geographical area of inner city Baltimore, 25-OH D levels are much lower among African-Americans than whites, identifying black preschool children as preferentially at risk for 25-OH D deficiency. Recent recommendations from the Institute of Medicine have challenged the traditional cut-offs for 25-OH D, suggesting that levels above 20 ng/mL instead of 30 ng/mL should be considered sufficient [25]. If we consider that suggested threshold, almost a quarter of subjects in our cohort would still be considered deficient. It is still uncertain what levels constitute sufficiency in asthma and other conditions apart from bone health; nevertheless, regardless of which guidelines are used to determine the optimal 25-OH vitamin D level, our study demonstrates that Baltimore, with one of the highest rates of asthma, is representative of a greater national epidemic of 25-OH D insufficiency in black children.

The findings from this study suggest that, although UV light is necessary for dermal vitamin D production, black urban children with asthma appear to be unresponsive to what little daily sunlight they do receive, as higher daily levels of outdoor exposure were not linked to higher 25-OH D. A limited number of other studies have also examined the relationship between sun exposure and 25-OH D, but little attention has been paid to this relationship in children, particularly African-Americans with asthma. Our study is unique in that it investigates this relationship between outdoor exposure and vitamin D status in a predominantly black population of preschool children, a group that includes children with the nation’s highest levels of asthma. In prior studies of healthy people, controlled UVB exposure increased serum 25-OH D in a dose-related manner [26,27]; however, African-Americans and Caucasians have a notable difference in this dose response. Scragg and Camargo demonstrated that in a racially-mixed population, non-Hispanic blacks had only modest increases in 25-OH D with increasing outdoor activity when compared to the dramatic changes observed in whites, with changes in serum 25-OH D observed only in response to extreme frequencies of outdoor activity (> 31 times in the past month) [28]. Taken with our findings, we conclude that cutaneous synthesis alone during limited outdoor exposure is not sufficient to raise serum levels effectively in black urban children. Exposure to sunlight beyond this range may cross a threshold where 25-OH D production begins to increase, as previous studies have shown that in healthy adults, higher doses of artificial UV are needed in darker-skinned individuals to measurably raise 25-OH D levels, compared those with lighter-skin [26]. However, a true prescription for natural UV exposure to achieve sufficient serum levels in black asthmatic children is yet to be determined.

Similarly, the predominantly African-American children in our study did not show any variation in 25-OH D levels with respect to season, in contrast to prior studies of predominantly-Caucasian populations whose levels are lowest in winter and highest in summer [29]. The observed differences in African-Africans may reflect a blunted response to the varying sunlight availability across seasons due to the diminished capacity of darker skin to synthesize vitamin D. We hypothesize that the lack of improvement in vitamin D status with increasing sun exposure—whether over a period of a day or across seasons—is a feature of increased skin pigmentation compounded by sub-optimal sun exposure. Spending a modest amount of additional time outside in regions with similar UV light availability is not likely an effective strategy to improve vitamin D status. On the other hand, promoting extreme doses of exposure, which may eventually lead to an improvement in levels, may not be practical or desired due to other risks of UV exposure. Alternative strategies to ensure adequacy through an enriched diet or dietary supplementation [8,30] may be necessary to compensate for such deficiencies.

Finally, our study supplements the small number of observational studies that show an association between personal 25-OH D and allergy and asthma morbidity [12-15,31]. Within our population, those children with lower 25-OH D levels had higher IgE levels. This inverse association has been shown previously [12], adding support to an allergy-mediated mechanism behind vitamin D’s effects. Furthermore, higher 25-OH D levels tended to be protective against asthma symptoms, including wheezing/coughing/chest tightness, symptoms while exercising, and cough without a cold or flu, although associations were not statistically significant. More studies, including those that are designed to address causal links, are needed to solidify a link between asthma outcomes and low 25-OH D levels. The 2011 Institute of Medicine review of the literature regarding dietary requirements of calcium and vitamin D concluded that there was insufficient evidence to inform recommendations on intake for extra-skeletal outcomes, and that further trials were needed to assess the benefit of supplementation on extra-skeletal health [25]. If found to be significant, improving individual vitamin D status could help protect against common urban asthma triggers in vulnerable children and result in a sustainable decrease in asthma morbidity.

One limitation of our study is that it was conducted at a single site in an urban area at a specific solar zenith angle, which typically receives less UV radiation than more equatorial regions of the world. However, it appears that astronomical factors may be less influential in determining vitamin D status, given that studies conducted in latitudes with higher UV radiation (e.g., Costa Rica, Georgia, Arizona) [12,32,33] show surprisingly similar rates of deficiency. Rather, lifestyle and behavioral factors may be more significant in an increasingly urbanized world. While our study did not record sunscreen use or protective clothing, or allow for direct measures of UV exposure which could vary with time of day and weather conditions, more generalizable features such as time spent outdoors and skin pigmentation may instead be the key determinants of vitamin D status and, in turn, asthma control. Additionally, rates of vitamin D supplementation and dietary intake of D-rich foods were not specifically assessed here. Finally, as with most other studies investigating vitamin D’s role in asthma, our design was cross-sectional, thus limiting our ability to establish a causal link between vitamin D and asthma morbidity. Future clinical trials are necessary to determine if vitamin D truly has the effects upon asthma health suggested by this observational literature.

## Conclusions

Our study showed that vitamin D insufficiency is common in highly affected African-American preschool-aged children with asthma, and that increases within the range of time typically spent outdoors by urban children would not be an effective strategy for repletion. Our results also add to the accumulating evidence of a link between lower 25-OH vitamin D levels and the allergic/asthmatic phenotype. Together, these findings offer that a combination of limitations in sunlight exposure and darker pigmentation may amplify the risk of vitamin D deficiency among black urban children, contributing to greater asthma morbidity than their white counterparts. Clinical trials are needed to determine whether augmentation of serum levels can attenuate asthma morbidity, and our results suggest that such trials may need to rely on dietary supplementation.

## Abbreviations

25-OH D: 25-hydroxyvitamin D; ng/mL: Nanograms per milliliter; NHANES: National health and nutrition examination survey; UV: Ultraviolet

## Competing interest

The authors declare that they have no competing interests.

## Authors’ contribution

SB, PB, and RR contributed to the design of the study questions and SB drafted the manuscript. MCM, NNH, EM, GD participated in the design of the study and contributed to the preparation of the manuscript. RP and JCB participated in the statistical analysis and interpretation. All authors read and approved the final manuscript.

## References

[B1] National center for health statistics, national center for health statistics-FASTATS 2006 http://www.cdc.gov/nchs/fastats/asthma.htm

[B2] Aligne CA Auinger P Byrd RS Weitzman M Risk factors for pediatric asthma. Contributions of poverty, race, and urban residence Am J Respir Crit Care Med 2000 162 873 877 10.1164/ajrccm.162.3.9908085 10988098

[B3] Ginde AA Liu MC Camargo CA Jr Demographic differences and trends of vitamin D insufficiency in the US population 1988–2004 Arch Intern Med 2009 169 626 632 10.1001/archinternmed.2008.604 19307527 PMC3447083

[B4] Saintonge S Bang H Gerber LM Implications of a new definition of vitamin D deficiency in a multiracial US adolescent population: the national health and nutrition examination survey III Pediatrics 2009 123 797 803 10.1542/peds.2008-1195 19255005

[B5] Kumar J Muntner P Kaskel FJ Hailpern SM Melamed ML Prevalence and associations of 25-hydroxyvitamin D deficiency in US children: NHANES 2001–2004 Pediatrics 2009 124 362 370 10.1542/peds.2009-0051 PMC3749840 19661054

[B6] Webb AR Who, what, where and when-influences on cutaneous vitamin D synthesis Prog Biophys Mol Biol 2006 92 17 25 10.1016/j.pbiomolbio.2006.02.004 16766240

[B7] Calvo MS Whiting SJ Barton CN Vitamin D fortification in the united states and Canada: current status and data needs Am J Clin Nutr 2004 80 6 Suppl 1710S 1716S 15585792 10.1093/ajcn/80.6.1710S

[B8] Moore CE Murphy MM Holick MF Vitamin D intakes by children and adults in the United States differ among ethnic groups J Nutr 2005 135 2478 2485 16177216 10.1093/jn/135.10.2478

[B9] Klepeis NE Nelson WC Ott WR Robinson JP Tsang AM Switzer P Behar JV Hern SC Engelmann WH The national human activity pattern survey (NHAPS): a resource for assessing exposure to environmental pollutants J Expo Anal Environ Epidemiol 2001 11 231 252 10.1038/sj.jea.7500165 11477521

[B10] Holick MF Resurrection of vitamin D deficiency and rickets J Clin Invest 2006 116 2062 2067 10.1172/JCI29449 16886050 PMC1523417

[B11] Holick MF Vitamin D deficiency N Engl J Med 2007 357 266 281 10.1056/NEJMra070553 17634462

[B12] Brehm JM Celedon JC Soto-Quiros ME Avila L Hunninghake GM Forno E Serum vitamin D levels and markers of severity of childhood asthma in Costa Rica Am J Respir Crit Care Med 2009 179 765 771 10.1164/rccm.200808-1361OC 19179486 PMC2675563

[B13] Brehm JM Schuemann B Fuhlbrigge AL Hollis BW Strunk RC Zeiger RS Serum vitamin D levels and severe asthma exacerbations in the childhood asthma management program study J Allergy Clin Immunol 2010 126 52 58 10.1016/j.jaci.2010.03.043 20538327 PMC2902692

[B14] Searing DA Zhang Y Murphy JR Hauk PJ Goleva E Leung DY Decreased serum vitamin D levels in children with asthma are associated with increased corticosteroid use J Allergy Clin Immunol 2010 125 995 1000 10.1016/j.jaci.2010.03.008 20381849 PMC2866800

[B15] Sutherland ER Goleva E Jackson LP Stevens AD Leung DY Vitamin D levels, lung function, and steroid response in adult asthma Am J Respir Crit Care Med 2010 181 699 704 10.1164/rccm.200911-1710OC 20075384 PMC2868500

[B16] Litonjua AA Childhood asthma may be a consequence of vitamin D deficiency Curr Opin Allergy Clin Immunol 2009 9 202 207 10.1097/ACI.0b013e32832b36cd 19365260 PMC2897155

[B17] Majak P Olszowiec-Chlebna M Smejda K Stelmach I Vitamin D supplementation in children may prevent asthma exacerbation triggered by acute respiratory infection J Allergy Clin Immunol 2011 127 1294 1296 10.1016/j.jaci.2010.12.016 21315433

[B18] CDC Asthma prevalence, health care use and mortality: United States, 2003–05 2010 http://www.cdc.gov/nchs/data/hestat/asthma03-05/asthma03-05.htm17947969

[B19] Maryland asthma control program. Asthma profile, Baltimore city. 2009 2009 http://fha.dhmh.maryland.gov/mch/documents/asthma_control/Profile_BaltimoreCity.pdf

[B20] Holick MF Vitamin D status: measurement, interpretation, and clinical application Ann Epidemiol 2009 19 73 78 10.1016/j.annepidem.2007.12.001 18329892 PMC2665033

[B21] Bischoff-Ferrari HA Giovannucci E Willett WC Dietrich T Dawson-Hughes B Estimation of optimal serum concentrations of 25-hydroxyvitamin D for multiple health outcomes Am J Clin Nutr 2006 84 18 28 16825677 10.1093/ajcn/84.1.18

[B22] Morgan W Crain EF Gruchalla RS O’Connor GT Kattan M 3rd Evans R Stout J Malindzak G Smartt E Plaut M Walter M Vaughn B Mitchell H Inner-City Asthma Study Group Results of a home-based environmental intervention among children with asthma NEJM 2004 351 1058 1080 10.1056/NEJMoa032097 15356304

[B23] Diggle P Heagerty P Liang K Zeger S Analysis of longitudinal data 2002 2 Oxford, England: Oxford University Press

[B24] Dezateux C Stocks J Lung development and early origins of childhood respiratory illness Br Med Bull 1997 53 40 57 10.1093/oxfordjournals.bmb.a011605 9158283

[B25] Institute of Medicine Dietary reference intakes for calcium and vitamin D 2011 Washington, DC: The National Academies Press 21796828

[B26] Armas LA Dowell S Akhter M Duthuluru S Huerter C Hollis BW Lund R Heaney RP Ultraviolet-B radiation increases serum 25-hydroxyvitamin D levels: The effect of UVB dose and skin color J Am Acad Dermatol 2007 57 588 593 10.1016/j.jaad.2007.03.004 17637484

[B27] Chen TC Chimeh F Lu Z Mathieu J Person KS Zhang A Kohn N Martinello S Berkowitz R Holick MF Factors that influence the cutaneous synthesis and dietary sources of vitamin D Arch Biochem Biophys 2007 460 213 217 10.1016/j.abb.2006.12.017 17254541 PMC2698590

[B28] Scragg R Camargo CA Jr Frequency of leisure-time physical activity and serum 25-hydroxyvitamin D levels in the US population: Results from the third national health and nutrition examination survey Am J Epidemiol 2008 168 577,86 discussion 587–91 18579538 10.1093/aje/kwn163 PMC2727193

[B29] Stryd RP Gilbertson TJ Brunden MN A seasonal variation study of 25- hydroxyvitamin D3 serum levels in normal humans J Clin Endocrinol Metab 1979 48 771 775 10.1210/jcem-48-5-771 429522

[B30] Harris SS Vitamin D and African Americans J Nutr 2006 136 1126 1129 16549493 10.1093/jn/136.4.1126

[B31] Freishtat RJ Iqbal SF Pillai DK Klein CJ Ryan LM Benton AS Teach SJ High prevalence of vitamin D deficiency among inner-city African American youth with asthma in Washington, DC J Pediatr 2010 156 948 952 10.1016/j.jpeds.2009.12.033 20236657 PMC3328513

[B32] Cole CR Grant FK Tangpricha V Swaby-Ellis ED Smith JL Jacques A Chen H Schleicher RL Ziegler TR 25-hydroxyvitamin D status of healthy, low-income, minority children in Atlanta, Georgia Pediatrics 2010 125 633 639 10.1542/peds.2009-1928 20351012 PMC2857317

[B33] Jacobs ET Alberts DS Foote JA Green SB Hollis BW Yu Z Martinez ME Vitamin D insufficiency in southern Arizona Am J Clin Nutr 2008 87 608 613 18326598 10.1093/ajcn/87.3.608 PMC4113473

